# PSMG3‐AS1 enhances glioma resistance to temozolomide via stabilizing c‐Myc in the nucleus

**DOI:** 10.1002/brb3.2531

**Published:** 2022-04-05

**Authors:** Li Zhou, Xuming Huang, Yu Zhang, Jihui Wang, Haiyan Li, Haiwei Huang

**Affiliations:** ^1^ Department of Rehabilitation The First Affiliated Hospital of Guangdong Pharmaceutical University Guangzhou China; ^2^ Department of Pathology Guangdong University of Traditional Chinese Medicine Guangzhou China; ^3^ Department of Neurology The Third Affiliated Hospital of Sun Yat‐sen University Guangzhou China; ^4^ Department of Neurology The First Affiliated Hospital of Sun Yat‐sen University Guangzhou China

**Keywords:** chemotherapy resistance, c‐Myc, long non‐coding RNA (LncRNA), PSMG3‐AS1, temozolomide

## Abstract

Glioblastoma (GBM) is the main form of primary brain malignancies with a dismal prognosis partly due to its invasive growth and rapid relapse. GBM frequently developed resistance to current standard‐of‐care therapeutic modalities, including surgery, radiation and chemotherapy, of which temozolomide (TMZ) is the most widely used first‐line anti‐GBM drug. Despite the intense efforts of the past decades, the underlying mechanisms of GBM resistance to TMZ remain largely unclear. Here we show that the long noncoding RNA (lncRNA) PSMG3‐AS1 is significantly upregulated in GBM and its expression correlates with the grade of glioma, with the highest level observed in GBM (Grade IV glioma). We also demonstrated that PSMG3‐AS1 mediates the resistance of GBM to TMZ, as knockdown of PSMG3‐AS1 remarkably increased the sensitivity whereas overexpression of PSMG3‐AS1 in sensitive GBM cell line induced a resistance phenotype to TMZ. Mechanistically, PSMG3‐AS1 directly binds to c‐Myc and thus stabilizes c‐Myc in the nucleus to promote the survival of GBM cells under treatment of TMZ. Our data demonstrated an unreported role of PSMG3‐AS1 in TMZ resistance and provide a potential novel target to tackle TMZ resistance in GBM.

## Graphical abstract

The mechanisms unraveled in this study might provide novel targets to overcome resistance to TMZ and bring benefits to GBM patients. The identification of PSMG3‐AS1 would also be beneficial to the pathological determination of resistance of GBM to TMZ in clinic

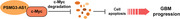

## 1 INTRODUCTION

Glioblastoma (GBM) is recognized as the most common malignancy of human primary brain tumor, with increasing global incidence and mortality rates (Siegel et al., 2020). Despite significant advances in the treatment of other solid tumors, the prognosis of GBM remains dismal with a median survival of less than 16 months (Liang et al., 2020). The current standard therapies are still limited to surgical resection, radiation therapy, and chemotherapy (Tabatabai & Glioblastoma, 2019). The standard‐of‐care therapies are ineffective in bringing GBM patients benefits due to the therapeutic resistance (Tomar et al., 2020) and rapid tumor recurrence (Tan et al., 2020). Therefore, it is urgent to identify new targets that mediate the resistance to improve the anticancer efficacy.

Recently, increasing evidence has documented the crucial and diverse roles of noncoding RNAs (Wang et al., 2020), which constitute the majority of the transcribed genome and are involved in the pervasive transcription (Statello et al., 2021). It has been documented that only about 2% of the human genome codes for proteins, while about 70−90% for noncoding RNAs (Statello et al., 2021), of which lncRNAs are characterized to have more than 200 nucleotides in length and serve an essential role in a variety of biological processes (Yao et al., 2019), such as differentiation, metabolism, migration, and apoptosis (Nair et al., 2020; Yao et al., 2019). An increasing number of studies have demonstrated that lncRNAs can serve as binding partners of proteins to serve as scaffold for protein‐protein interaction or to stabilize proteins (Ribeiro et al., 2018). Previous studies have shown that lncRNAs could promote the resistance (Gao et al., 2021) and recurrence of GBM (Mazor et al., 2019; Zeng et al., 2017). As a lncRNA, PSMG3‐AS1 has been shown to play a crucial role in the malignancy of breast (Cui et al., 2020), liver (Zhang et al., 2020), and lung cancer (Yue et al., 2020). While PSMG3‐AS1 is reported to be upregulated in GBM and its overexpression distinguish GBM from Sarcoidosis (Chen et al., 2020), its role and the underlying mechanism in GBM remains to be explored.

The present study aimed to reveal the role of lncRNA PSMG3‐AS1 in GBM resistance to TMZ. The present study revealed that PSMG3‐AS1 was increased in glioma compared to normal brain tissues and GBM showed the highest expression level. The results also demonstrated that PSMG3‐AS1 expression was associated with the resistance to TMZ of GBM cells. Furthermore, PSMG3‐AS1 was shown to localize in the nucleus and regulate the expression of c‐Myc via direct RNA‐protein binding. Thus, PSMG3‐AS1 may act as a potential therapeutic target gene to increase the sensitivity of GBM to TMZ.

## 2 MATERIALS AND METHODS

### 2.1 Tumor samples

Human primary glioma samples and normal brain tissues were obtained from The First Affiliated Hospital of Guangdong Pharmaceutical University under the approval of the Ethical Committee. Informed consents were obtained from all patients prior to the surgical resection. Online analysis of GBM RNA‐seq data were retrieved from The Cancer Genome Atlas (TCGA) and analyzed on Gepia (http://gepia.cancer‐pku.cn/#survival) (Tang et al., 2017).

### 2.2 Plasmid construction

PSMG3‐AS1 overexpression plasmid used in this study was purchased from GenePharma (Shanghai, China). PSMG3‐AS1‐specific shRNA vector and nontargeting control (NC) were constructed following the previously reported rapid shRNA cloning method (Zhou et al., 2018) using the following shRNA sequence (5′‐AGTTCAGAATGGGAGACGTCC‐3′) to specifically knock down PSMG3‐AS1, and a nontargeting shRNA (5′‐ TTGGTGCTCTTCATCTTGTTG‐3′) (Zhou et al., 2019) as control.

### 2.3 Stable cell line establishment

Stable cell lines with PSMG3‐AS1 overexpressed or interfered were established using lentivirus as previously described (Zhou et al., 2018). Briefly, transgene plasmid was cotransfected with packaging plasmids (psPAX2 and pMD2.G) using PEI into HEK293T cells. The pseudovirus‐containing supernatant was harvested, filtered, and used to infect the target cells. Subsequently, puromycin was supplemented to the culture medium (2 μg/ml) for at least 7 days to achieve stable cell line establishment.

### 2.4 Cell viability assay and determination of IC50 to TMZ

Cell Counting Kit‐8 (CCK8, Sigma) was used to determine the cell viability and the 50% growth inhibitory concentration (IC50) of TMZ. Cells were seeded in 96‐well plates at a density of 5 × 10^3^ cells per well and were incubated for 72 h. Subsequently, TMZ was added to culture medium at final concentrations of 0, 20, 40, 60, 80, and 120 μM. Then, CCK8 was used following the manufacturer's recommendations to determine the cell viability. All experiments were performed in triplicate.

### 2.5 RNA extraction and quantitative real‐time polymerase chain reaction (qRT‐PCR)

Total RNA was extracted from the tissue samples or cells using RNAiso Plus (#9108, Takara, Beijing, China). The first strand cDNA was synthesized the HiScript® III 1st Strand cDNA Synthesis Kit (#R312, Vazyme, Nanjing, China). qRT‐PCR was performed using an AceQ Universal SYBR qPCR Master Mix (#Q511, Vazyme) on an ABI‐7300 instrument (Applied Biosystems, USA). All procedures were performed following the manufacturer's protocol. Relative expression levels were determined using the 2^−ΔΔCq^ method and normalized to β‐actin. The primer sequences used were as follows. PSMG3‐AS1 forward, 5′‐GAAGCAGAACCAACGCACAG‐3′ and reverse, 5′‐GCATAATCCAATCCCTCAAGAA‐3′; ACTB forward, 5′‐CCTGGCACCCAGCACAAT‐3′ and reverse, 5′‐GGGCCGGACTCGTCATAC‐3′.

### 2.6 Western blot

Total protein was isolated in cell lysis buffer for western blot analysis and immunoprecipitation (Beyotime, China). After quantification using a BCA kit (Thermo Fisher), equal amounts of protein lysates were separated on 10% SDS‐PAGE and transferred to 0.45 μm PVDF membranes, which were then blocked with blocking buffer at room temperature for 1 h. Subsequently, blots were incubated with primary antibodies at 4°C overnight with the following primary antibodies: anti‐c‐Myc (#18583, CST, 1: 1000) and anti‐β‐Actin (#4970, CST, 1: 2000). The blots were visualized using Western Chemiluminescence Substrate (Thermo Fisher, USA) on a chemiluminescence apparatus (Bio‐Rad, USA).

### 2.7 Statistical analysis

SPSS 18.0 software (SPSS, Inc.) was used for statistical analysis. Data are presented as the mean ± SEM of at least two independent experiments. Statistical differences between groups were analyzed using a Student's *t*‐test (two groups) and a χ^2^ test was used to determine the correlation between the expression of PSMG3‐AS1 and clinicopathological features. Only when *p* < .05, it was considered to indicate a statistically significant result.

## 3 RESULTS

### 3.1 PSMG3‐AS1 is upregulated in glioblastoma and correlates with GBM grades

**FIGURE 1:**
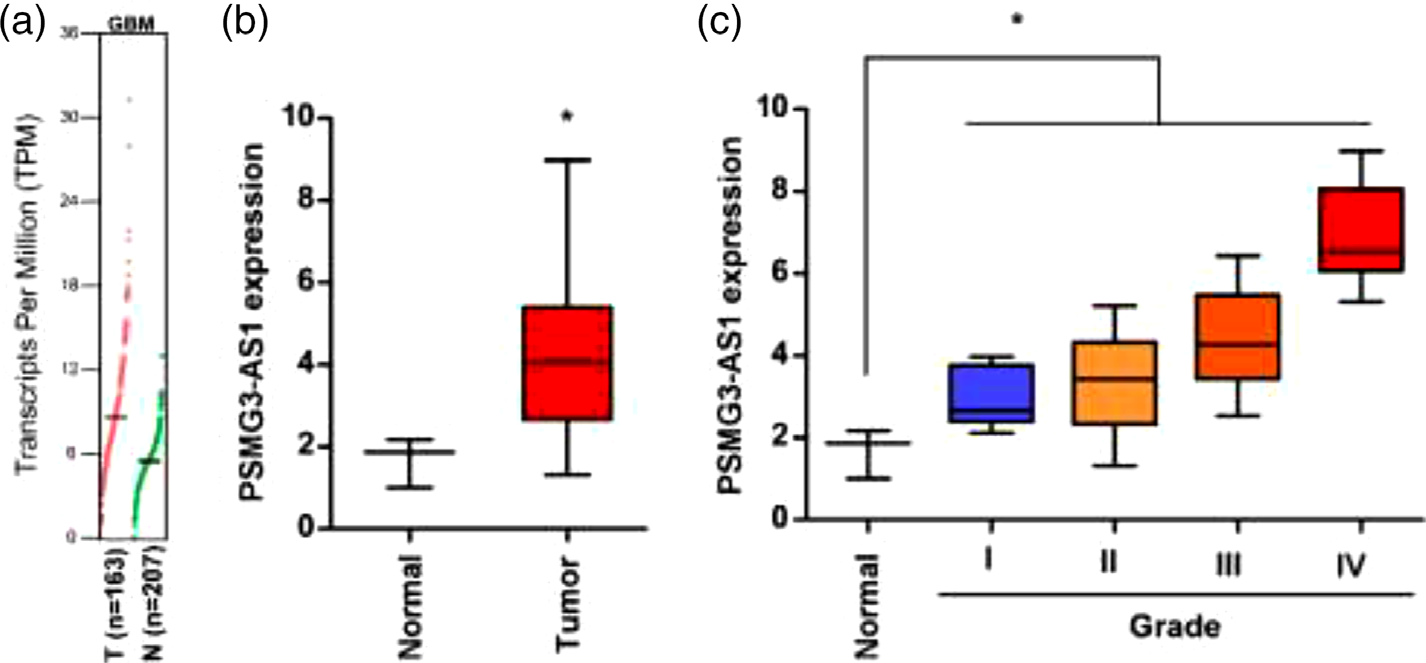
lncRNA PSMG3‐AS1 is overexpressed in glioma, showing a highest level in GBM. (a) Expression of PSMG3‐AS1 was analyzed to compare GBM tissue and normal brain tissues online at Gepia (http://gepia.cancer‐pku.cn/detail.php?gene = PSMG3‐AS1, *n* = 163 for tumor and *n* = 207 for normal). (b, c) The expression of PSMG3‐AS1 in normal and glioma tissues, where *n* = 4 in normal group, *n* = 20 in tumor group; *n* = 5 in Grade I, *n* = 10 in Grade II, *n* = 9 in Grade III and *n* = 6 in Grade IV as summarized in Table 1. *p < .05

To determine the expression pattern of PSMG3‐AS1, we analyzed the expression of PSMG3‐AS1 at both Gepia, we found that PSMG3‐AS1 expression level was significantly increased in tumor samples compared with normal cohort (Figure 1a), suggesting that PSMG3‐AS1 might play a role in GBM progression. Consistent with the public data, the same expression pattern was also observed in the samples collected in our hospital (Figure 1b), showing a significantly higher PSMG3‐AS1 level in tumors than normal tissues. To further assess the role of PSMG3‐AS1 in glioma, we plotted the expression pattern of PSMG3‐AS1 according to the grade of glioma. Interestingly, the expression of PSMG3‐AS1 is remarkably positively correlated with the grade of glioma, with the highest level in GBM (Figure 1c). These results indicated that PSMG3‐AS1 is likely to promote the progression of GBM.

### 3.2 HSMG3‐AS1 expression level is associated with resistance to temozolomide

**FIGURE 2:**
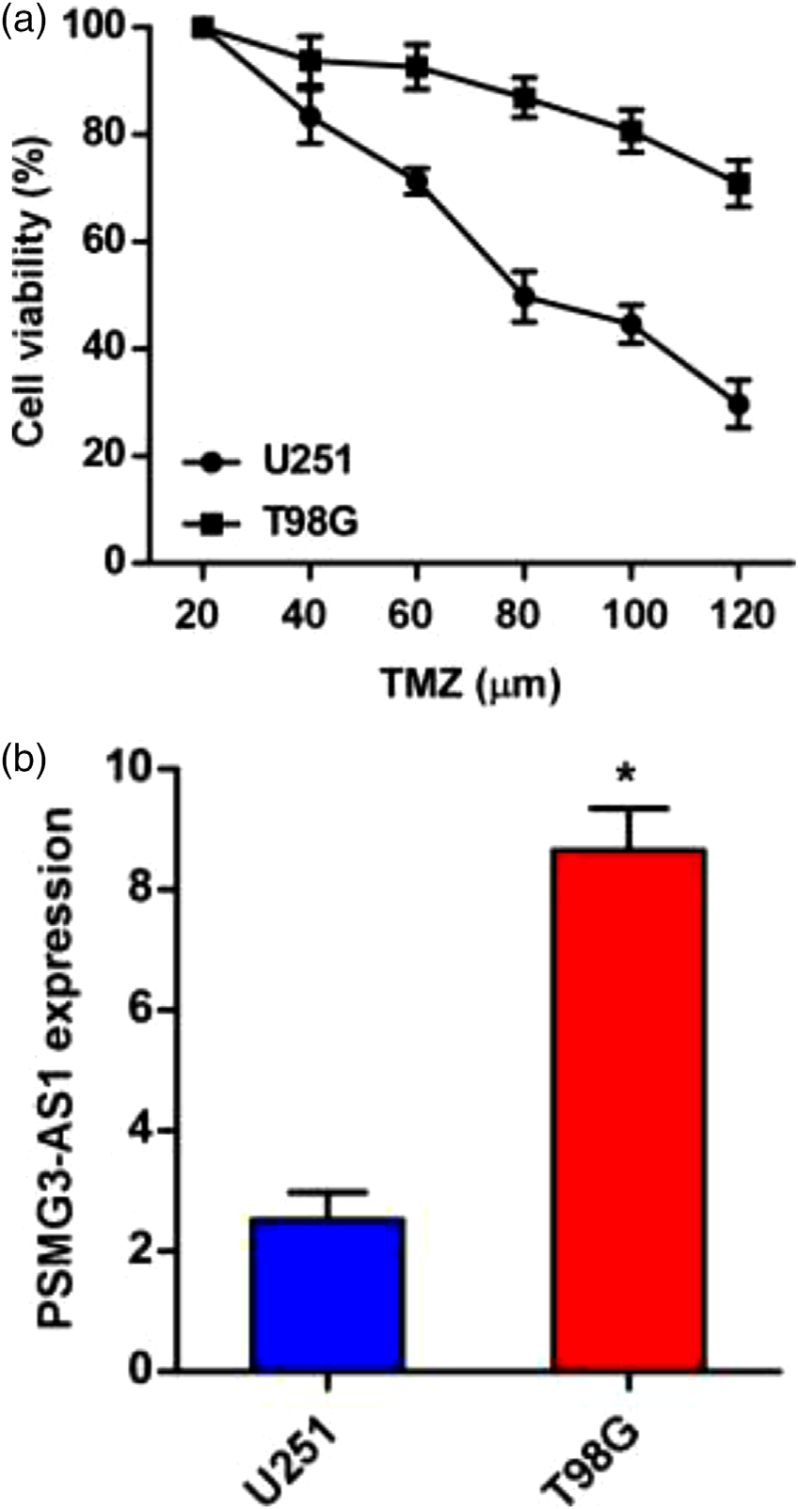
PSMG3‐AS1 expression level is associated with TMZ sensitivity. (a) The IC50 of U251 and T98G to TMZ was determined. (b) The expression of PSMG3‐AS1 was determined by qRT‐PCR, using U6 as internal control. *p < .05

As the most widely used first‐line chemotherapy drug, TMZ resistance in GBM is one of the main mechanisms of therapeutic failures. We treated GBM cell lines U251 and T98G with TMZ, and then determined the sensitivity of these cells to TMZ. It is obvious that U251 is more sensitive to TMZ than is T98G (Figure 2a). To reveal whether the resistance of T98G to TMZ was in some extent rendered by PSMG3‐AS1, we detected the level of PSMG3‐AS1 in both cell lines by qRT‐PCR. As expected, we found that T98G expressed significantly higher level of PSMG3‐AS1 than did U251 (Figure 2b). These data implied that PSMG3‐AS1 might be involved in the resistance to GBM to TMZ.

### 3.3 PSMG3‐AS1 promotes GBM resistance to temozolomide

**FIGURE 3:**
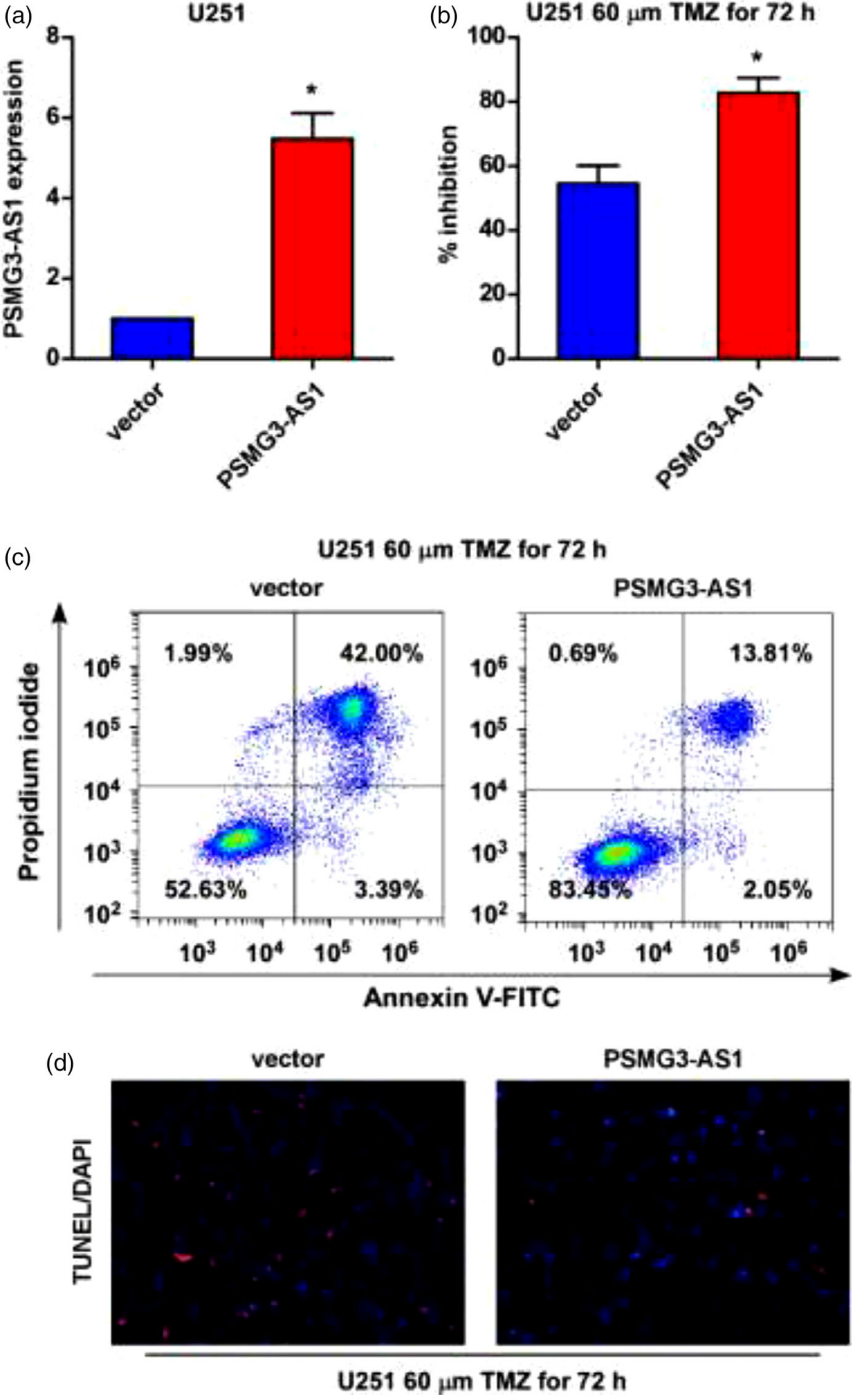
Overexpression of PSMG3‐AS1 in U251 enhanced TMZ resistance. (a) Overexpression of PSMG3‐AS1 in U251 was confirmed by qRT‐PCR with U6 as internal control. (b) The inhibition effect of TMZ was tested after PSMG3‐AS1 overexpression, where TMZ (60 μM) was added for 72 h. (c, d) Apoptosis was detected by Annexinv‐V/PI‐based flow cytometry and TUNEL staining, respectively, in PSMG3‐AS1 overexpression U251 after TMZ treatment for 72 h. *p < .05

**FIGURE 4:**
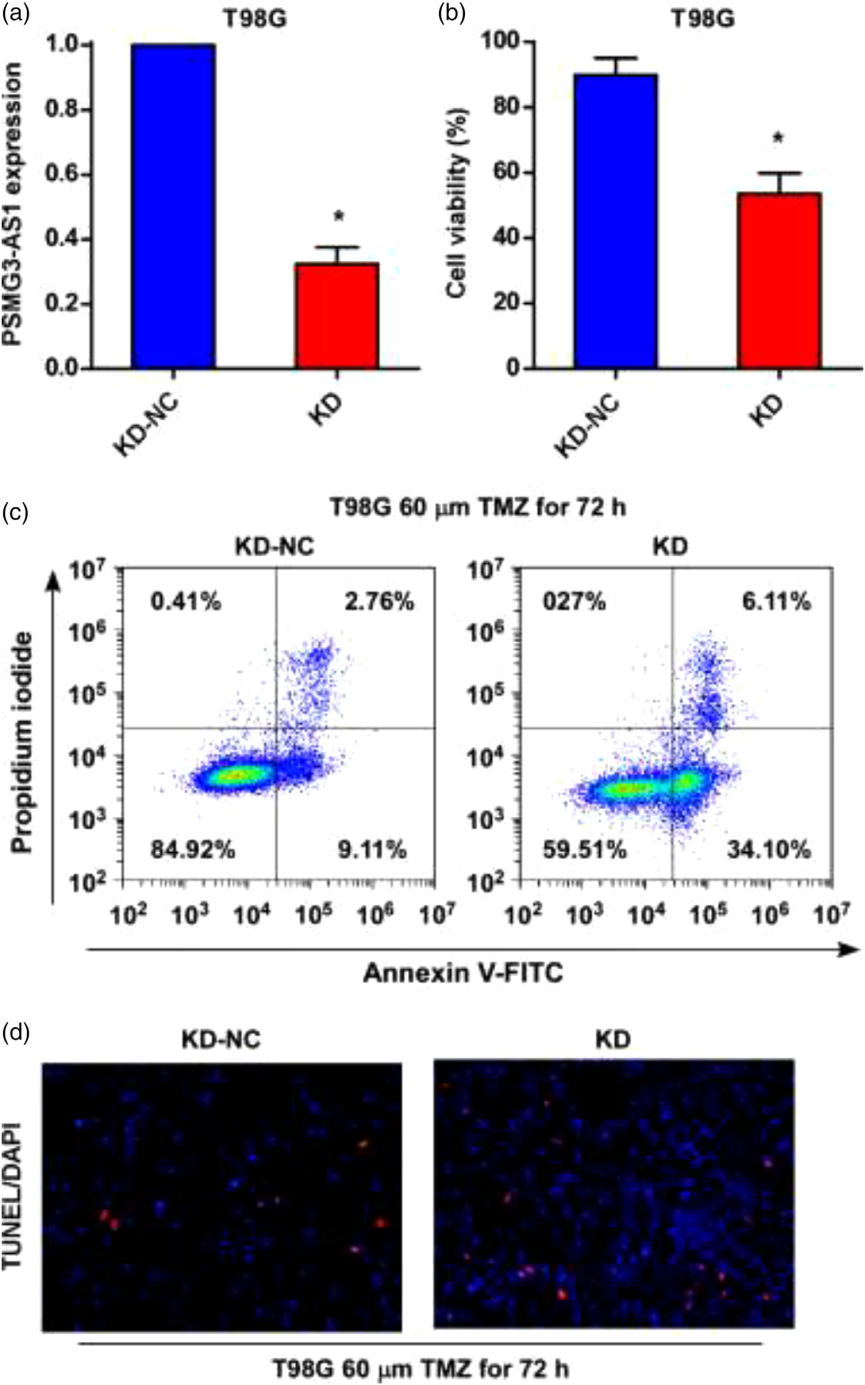
PSMG3‐AS1 knockdown sensitized T98G to TMZ treatment. (a) Knockdown of PSMG3‐AS1 in T98G was confirmed by qRT‐PCR with U6 as the internal control. (b) The inhibition effect of TMZ was determined after PSMG3‐AS1 knockdown, culturing with TMZ (60 μM) for 72 h. (c, d) Apoptosis was detected by Annexinv‐V/PI‐based flow cytometry and TUNEL staining, respectively, in PSMG3‐AS1 knockdown T98G cell after TMZ treatment for 72 h. *p < .05

To determine whether the expression of PSMG3‐AS1 increased the resistance of GBM to TMZ, we then overexpressed PSMG3‐AS1 in U251 (Figure 3a). After overexpression, the apoptosis of U251 upon TMZ treatment was significantly reduced as detected by both PI‐Annexin V staining (Figure 3b) and TUNEL staining (Figure 3c), indicating that PSMG3‐AS1 overexpression indeed increased the resistance to TMZ. On the other hand, when we silenced PSMG3‐AS1 in T98G by shRNA (Figure 4a), the resistance of T98G to TMZ was remarkably decreased, as demonstrated by increased apoptotic cells when TMZ was added (Figure 4b and c). Taken together, these data demonstrated that PSMG3‐AS1 actually increased the resistance of GBM to TMZ.

### 3.4 PSMG3‐AS1 is localized in the nucleus and stabilizes c‐Myc protein

**FIGURE 5:**
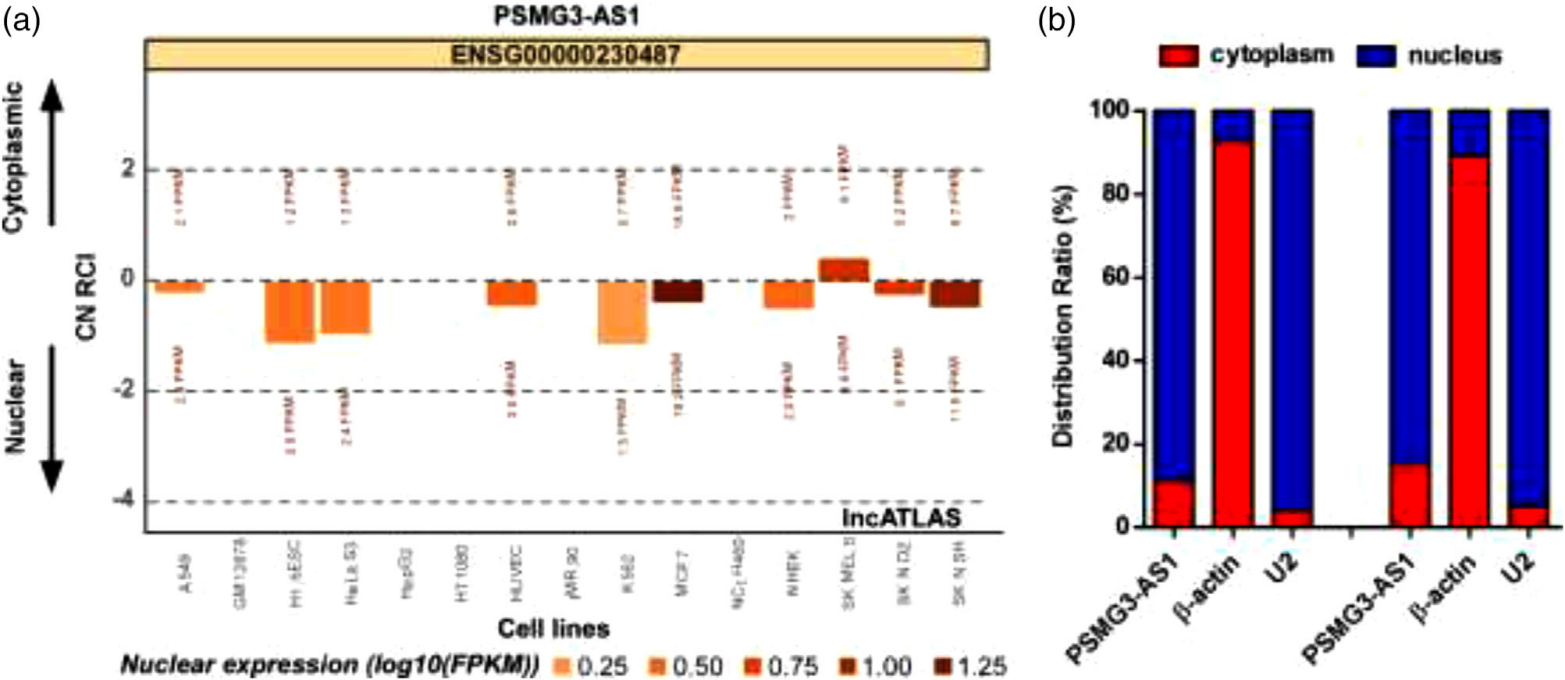
PSMG3‐AS1 is a nucleus localized lncRNA. (a) PSMG3‐AS1 was predicted to be enriched in the nucleus at lncATLAS. (b) The subcellular localization of PSMG3‐AS1 was determined by qRT‐PCR following nuclear/cytoplasmic RNA isolation, with U2 and β‐actin as representative nuclear and cytoplasmic RNA, respectively

**FIGURE 6:**
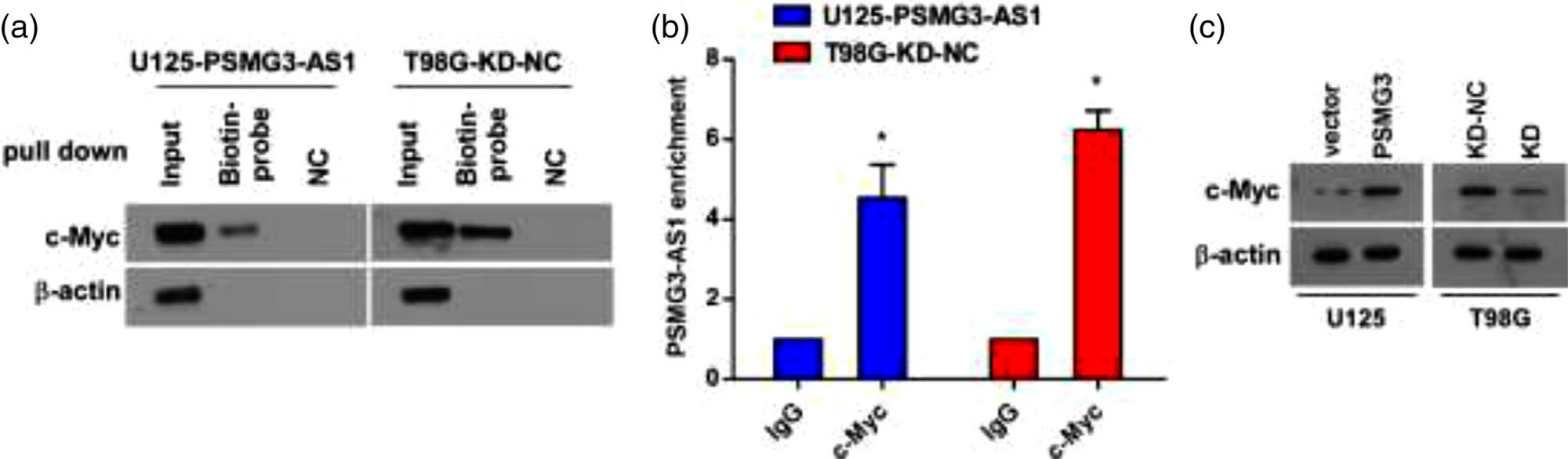
PSMG3‐AS1 interacts with c‐Myc and enhances its stability. (a) Biotin‐PSMG3‐AS1 was used to pull down c‐Myc, with β‐actin as loading control. (b) Immunoprecipitation was performed followed by qRT‐PCR to determine the binding of c‐Myc to PSMG3‐AS1, where isotype IgG was used as control. (c) Expression level of c‐Myc was positively correlated with the expression of PSMG3‐AS1. *p < .05

As the localization of molecules reflects their functions, we then determined the localization of PSMG3‐AS1 to further clarify its function at molecular level. As shown by the online tool lncATLAS, PSMG3‐AS1 was predicted to be localized in nuclear compartment (Figure 5a). To verify the predicted results, we separated the subcellular fractionations as nucleus and cytoplasm to determine the localization of PSMG3‐AS1. We found that PSMG3‐AS1 was indeed mainly in the nucleus (Figure 5b). As PSMG3‐AS1 was shown to mainly localize in the nucleus, it is like to interact with nuclear molecules to exert its function. It is well known that c‐Myc plays a vital role in GBM resistance to TMZ (Dang, 2012). To uncover whether there is a link between PSMG3‐AS1 and c‐Myc, we performed RNA pulldown assay. We observed that c‐Myc and PSMG3‐AS1 could pull down each other reciprocally (Figure 6a and b). These data indicated that PSMG3‐AS1 could probably mediate the resistance of GBM to TMZ via c‐Myc. Indeed, we observed that when PSMG3‐AS1 was overexpressed in U251, the c‐Myc protein was also increased, whereas PSMG3‐AS1 knockdown resulted in decreased c‐Myc protein. Taken together, these data demonstrated that PSMG3‐AS1 is probably associated with GBM resistance to TMZ via stabilizing c‐Myc in the nucleus.

## 4 DISCUSSION

Surgical resection followed by radiotherapy and chemotherapy is the current standard treatment for glioma (Tabatabai & Glioblastoma, 2019). It has been proven that effective chemotherapy can significantly improve the survival rate and survival time of patients with GBM (Siegel et al., 2020). However, acquired TMZ resistance is frequently observed and limits the efficacy of TMZ to recurrent GBM patients (Lee, 2020). Therefore, elucidating the underlying molecular mechanisms to block TMZ chemotherapy resistance is of great value to expand the range of therapeutic options and extend the survival of GBM patients.

The present study mainly investigated the functions of PSMG3‐AS1 in GBM resistance to TMZ, a frontline chemotherapeutic drug. We found that the expression of PSMG3‐AS1 were significantly upregulated in glioma tissues compared to normal tissues. Interestingly, its expression increases with the progression of glioma, reaching the highest level in GBM (WHO IV). PSMG3‐AS1 is also found to be expressed at higher level in TMZ resistant cell (T98G) than the sensitive cell (U251), and overexpression or knockdown of PSMG3‐AS1 reversed this phenotype. In addition, PSMG3‐AS1 is nucleus localized and stabilizes c‐Myc to confer resistance to TMZ.

The functionality of PSMG3‐AS1 has only been investigated in breast cancer (Cui et al., 2020), hepatocellular (Zhang et al., 2020), and lung cancer (Yue et al., 2020). Although PSMG3‐AS1 is reported to be overexpressed in GBM in contrast to Sarcoidosis (Chen et al., 2020), its role in GBM, especially to TMZ resistance, remains yet to be unraveled. It is observed that PSMG3‐AS1 is overexpressed in cancer cells and is involved in promoting the migration and proliferation of cancer cells via sponging microRNAs (Cui et al., 2020; Yue et al., 2020; Zhang et al., 2020). However, according to our data, PSMG3‐AS1 is mainly localized to nucleus where it is not likely to function as a sponge of microRNAs, hence other mechanisms are required to explain the function of PSMG3‐AS1. Our further work demonstrated that PSMG3‐AS1 is able to stabilize c‐Myc in the nucleus.

To the best of our knowledge, this study is the first to report the function of PSMG3‐AS1 in TMZ resistance of GBM, the most prevalent primary glioma. Our in vitro cell experiments showed that PSMG3‐AS1 enhances the resistance to TMZ and knockdown abolished this phenotype. Accordingly, interfering the expression of PSMG3‐AS1 or the interaction between PSMG3‐AS1 and c‐Myc could serve as a novel therapeutic opportunity to conquer the resistance to TMZ in GBM.

## 5 CONCLUSION

Conclusively, our data showed that lncRNA PSMG3‐AS1 is overexpressed in GBM compared to normal brain tissues and its expression is positively correlated with TMZ resistance. The underlying mechanism by which PSMG3‐AS1 enhances the resistance to TMZ is to stabilize c‐Myc via directly binding. Our research highlighted the role of lncRNA in TMZ resistance of GBM and provided a potential target to sensitize GBM to TMZ.

## AUTHOR CONTRIBUTIONS

L.Z. and H.‐W.H. contributed to the conceptual design of the study and drafted the manuscript. L.Z., Z.Y., X.‐M.H., Y.‐Z., J.‐H.W., and H.‐Y.L. were involved in experiment conduction, data acquisition, and data analysis. L.Z. and H.‐W.H. drafted the manuscript.

## CONFLICT OF INTEREST

All authors declare no conflict of interest.

## FUNDING

This work was supported by National Natural Science Foundation of China (82002388 to L.Z.).

### PEER REVIEW

The peer review history for this article is available at https://publons.com/publon/10.1002/brb3.2531.

## Supporting information



Supporting Information

## Data Availability

The datasets used and analyzed in the current study are available from the corresponding author in response to reasonable requests.
