# Supplementary material for: PSMG3‐AS1 enhances glioma resistance to temozolomide via stabilizing c‐Myc in the nucleus
Source: Brain Behav. 2022 Apr 5;12(5):e2531. doi: 10.1002/brb3.2531 (PMC9120888; doi:10.1002/brb3.2531)
Supplement: Supplementary file 1 — Supporting Information [file BRB3-12-e2531-s001.docx]

**Table 1.** Clinicopathological characteristics of studied patients in 30 glioma specimens

| Characteristics | No. of Cases | % |
| --- | --- | --- |
| **Age (years)** |  |  |
| ≤45 | 21 | 70.00 |
| >45 | 9 | 30.00 |
| **Gender** |  |  |
| Male | 19 | 63.33 |
| Female | 11 | 36.67 |
| **WHO grade** |  |  |
| I | 5 | 16.67 |
| II | 10 | 33.33 |
| III | 9 | 30.00 |
| IV | 6 | 20.00 |
| **Vital Status (at follow-up)** |  |  |
| Alive | 12 | 40.00 |
| Dead | 18 | 60.00 |
